# Book Review

**Published:** 1929

**Authors:** 


### Reviews of Books

**Ker's Infectious Diseases.** By C. Rundle, M.D., M.R.C.S., L.R.C.P., D.P.H. Third edition. Pp. xii., 614. 32 illustrations. London: Oxford University Press. 1929. Price 30s.—This edition has been brought thoroughly up to date by Dr. Claude Rundle, and in some parts has been entirely re-written. There is so much new matter in the book that the chapter on Relapsing Fever has been omitted. Much recent work on the ætiology of infectious diseases has been added, and the different methods of treatment are admirably and clearly set forth. The illustrations are very numerous and particularly good. It is a work which can be very confidently recommended both to students and practitioners.

**Contributions to Psychiatry, Neurology and Sociology.** (The Mott Memorial.) Edited by J. R. Lord, M.D., F.R.C.P.E. Pp. xiv., 401. Illustrated. London: H. K. Lewis & Co. Ltd. 1929. Price 21s. This is a compilation of a large number of contributors, most of whom are either distinguished psychiatrists or neurologists. The appeal of the book will, however, be chiefly to those interested in psychiatry. There is naturally a tendency for the various writers to choose subjects which had a special appeal to Sir Frederick Mott. This is particularly noticeable in a closely-reasoned article by Mapother in his plea for accuracy in assessment of alcoholic morbidity. The chapter on the prevention and early treatment of mental disorders by Sir Hubert Bond gives prominence to the urgent need for alteration in our Lunacy Laws, while Dr. Helen Boyle's contribution on the early treatment of border-line patients is a welcome supplement to the subject. On the scientific side Winkler contributes a most interesting article on the physiology of the mammalian cerebellum and Elliot Smith has an excellent chapter on the anatomy of the visual cortex in man. Bernard Hart writes a helpful chapter on the ætiology of alcoholism. Readers who are interested in psychological studies of dramatists will find a fascinating contribution by Jelliffe on Ibsen, whom he terms "the apostle of the psychopath." A chapter which deserves special mention is that by Geary, which gives, perhaps, the best account that we have so far on the histopathology of general paralysis treated by malaria. Throughout the book all the articles are so good, that it is, perhaps, invidious to single out any chapters for special notice.

**Alcohol and Human Life.** By C. C. WEEKS, M.R.C.S. Pp. xii., 201. London: H. K. Lewis & Co. Ltd. 1929. Price 3s. 6d.—Dr. Courtenay C. Weeks is to be congratulated on the successful way in which he has revised and re-edited Horsley and Sturge's well-known text-book. During recent years there has been a lull in the teaching of the facts about alcohol and its effects on the individual and on the national life, but with an annual drink bill of £298,000,000 there is much yet to be done. This monograph will prove an invaluable work of reference to temperance teachers, and particularly should be in the hands of all such as have the training of medical students and nurses. The subject is treated in a broad and fair spirit, and although replete with statistics, the book is eminently readable. As Sir Thomas Barlow says in the foreword, "It will repay careful perusal and frequent reference."

**Some Principles of Minor Surgery.** By V. Z. COPE, M.S., M.D., F.R.C.S. Pp. xii., 159. 82 illustrations. London: Oxford University Press. 1929. Price 10s. 6d.—This book has broken away from the traditional ones on minor surgery. The author has not hesitated to throw overboard oft-repeated but out-of-date methods of treatment. He has placed his principles first, and from them worked out his line of treatment. This makes very interesting and refreshing reading. On antiseptics the author has a good word for only two, namely flavine and eusol. The Carrel-Dakin method of using the latter is carefully described. The treatment of inflammation and acute abscess is fully dealt with, the diagrams being clear and easy to follow. A special chapter is devoted to the important subject of infections of the hand, and here Kanavel's teaching is closely followed. In the chapter on sprains the modern ideas on tennis elbow are stated, but the author does not give his personal views. The article on ambulatory fractures is excellent, but we are surprised to read that the abduction splint for fractures of the upper end of the humerus is unnecessary. In the treatment of Colles's fracture the author describes his modification of Carr's splint. It is made of pliable metal, and allows the wrist to be put up in either flexion or extension; it is also reversible and can be used for either hand. The extremely important subject from the practitioner's point of view of acute retention is dealt with in a practical manner, the illustrations here again being a great help. The final chapter deals with minor operations, including the injection of piles and varicose veins. We can strongly recommend this book to the general practitioner for its clear conception and modern views of the principles and treatment of minor surgery.

**On Prescribing Physical Treatment.** By M. B. RAY, D.S.O., M.D. Pp. x., 179. Illustrated. London: William Heinemann Ltd. 1929. Price 10s. 6d.—The preface states that "this book is written entirely from the standpoint of the prescriber," and that "physical treatment implies utilization of all kinds of heat and cold, all electrical currents, all forms of radiant energy, such as radium, X-rays, ultra-violet rays, visible rays of the sun, manipulations, exercises, etc." Yet no further mention is made of radium, X-rays, exercises or manipulations (except under douches). About half the book is on hydrotherapy and thermotherapy, six pages on massotherapy, the rest on electrotherapy and actinotherapy; climatotherapy is hardly mentioned. Still there is a large amount of information condensed into the small space, and most of it is comprehensive and accurate, though there are one or two slips, such as, on page 34, Nauheim baths "beginning at fifteen minutes"; artificial brine baths made with only 6 lb. to 40 gallons of water (p. 35); Turkish baths cause no rise in body temperature (p. 67) although radiant heat does (p. 73); "hyperæmia more pronounced at anode" (p. 104), though the "positive pole acts as vaso-constrictor" (p. 106); the bath used for electric water baths should *not* be in "excellent contact with earth," as stated on pp. 125 and 128. The usefulness of the book would have been enhanced if such treatments as Aix and Vichy douches, Turkish and general radiant heat baths, ionization and diathermy and so on had been compared and contrasted more fully; also the combining of treatments, as radiant heat and ionic medication, Turkish baths and ultra-violet light, etc. Again, a fuller index of injuries and diseases known to benefit by physiotherapy would have been of great assistance to the general practitioner. The book is illustrated by half a dozen well-known photographs from Bath and a few diagrams.

**Technique of Gastroscopy.** By W. STERNBERG (Berlin). Pp. 18. London: John Bale, Sons and Danielsson Ltd. 1929. Price 3s. 6d.—This little brochure gives almost in tabular form the salient points of the author's technique. Except that he relies on palpation rather than on vision, it is not clear how this differs essentially from routine methods. The English is not uniformly felicitous *e.g.* "I regard the avoidance of the all-narcotics as essentially a thankful procedure for abolishing any danger." Keeping in mind this scorn of anæsthesia, local or general, one wonders whether his observation that "it is easier to perform gastroscopy on women and children than on vigorous men" is because he "considers it is wrong to tie the patient down!"

**A Graphic Guide to Elementary Surgery.** By TH. NAEGELI (Bonn). Translated by J. SNOWMAN, M.D., M.R.C.P. Pp. viii., 206. Illustrated. London : John Bale, Sons & Danielsson Ltd. 1929. Price 12s. 6d.—We are very pleased to welcome this English edition of Professor Naegeli's book. It consists of a short description of surgical principles, accompanied by many very clear and striking diagrams. For example, in describing the course and treatment of a pulmonary cavity, two diagrams, each including a temperature chart, a sketch of the lung, a measured receptacle for sputum and a microscopic slide of the sputum serve to show the effects of good treatment. It will be an admirable little book for dressers as an introduction to ward work and for nurses. The same principle of numerous diagrammatic illustration might with advantage be adopted for a larger text-book of surgery.

**Hemorrhoids.** By A. S. MORLEY, F.R.C.S. Fourth Edition. Pp. x., 122. Illustrated. London : Oxford University Press. 1929. Price 6s.—We favourably reviewed the first edition of this well-known book. It contains an excellent description of hemorrhoids and of the principles and technique of their treatment by interstitial injections. With regard to the usual operations for piles, not here described, the author writes : " I am convinced that operation is rarely necessary, and that the condition may be dealt with as satisfactorily and as safely by the method of interstitial injections." This opinion is expressed after an experience of more than 3,000 cases in which injections were used. In an appendix to the present edition the author describes a technique which he considers superior to that used until 1926. No one who uses the injection method in the treatment of hemorrhoids can afford to ignore this book.

**The Medical Museum.** By S. H. DAUKES, M.D. D.P.H. Pp. 183. Illustrated. London : The Wellcome Foundation Ltd. 1929.—Dr. Daukes has done useful service in compiling this booklet of museum work. It shows how the museum may be used for teaching purposes. Pictures, diagrams and many other accessories are subsidized to illustrate diseases and specimens. To look through the book brings back to one's mind the Dresden Hygiene Exhibition of 1911. As a matter of fact, some of the pictures are copies taken from the Deutsches Hygienisches Museum at Dresden. There is a very useful article on methods of preservation and preparation. We congratulate the author on his meritorious work and express our thanks to the Foundation for its most acceptable publication.

**The Treatment of Varicose Veins by Intravenous Injections.** By J. P. D. McLATCHIE, M.D. Pp. viii., 52. Illustrated. London: William Heinemann, Ltd. 1928. Price 3s. 6d.—This small book presents a concise yet sufficiently detailed account of the treatment of varicose veins by injecting into them certain substances which cause thrombosis and subsequent obliteration of their lumen. It opens with a few interesting pages on the historical aspect of the remedy, which remind us that the treatment is not new, but was practised some eighty years ago with varying success. The author mentions all the preparations of any importance which have been used for this purpose, and considers in detail all the pros and cons of those of any proved therapeutic value. The dosage, technique, effects, complications, indications and contra-indications for this method of treatment receive their due share of publicity. The book is well worth perusal by any who contemplate the adoption of this method of treatment, and will save much time in searching medical literature for details here given in thirty-nine small pages.

**An Inquiry into Post-Operative Tetanus.**—A Report to the Scottish Board of Health. By T. J. MACKIE M.D., D.P.H. H.M. Stationery Office. Price 1s.—Professor Mackie was asked to report upon the occurrences of post-operative tetanus and to inquire into the effectiveness of the means employed for the sterilization of catgut. The brochure contains an interesting account of cases of post-operative tetanus and discusses the possibility of contamination by tetanus bacilli. Tetanus bacillus occurs as a commensal organism in the intestinal tract of herbivorous animals, and the sheep's intestine—from which surgical catgut is largely prepared—is therefore to be regarded as a potential source of these organisms. Cleansing and sterilization of the material presents many difficulties for the manufacturer, and Dr. Mackie demonstrates the great differences in bactericidal power of possible disinfecting agents. It appears that hydrogen peroxide and iodine trichloride are among the more effective substances. Further, even for hydrogen peroxide the minimum contact of twelve hours is required, and it is recommended that the ribbons of intestine which are spun into strands ought to undergo further sterilization in iodine for fourteen days. The report emphasizes the necessity for frequent bacteriological tests on batches of catgut used in operative work. For purposes of reference it ought to find a place on every surgeon's shelf.

**Cancer and Cancer Research.** Manchester: Sherratt and Hughes. 1928. Price 1s. 6d.—During the spring of 1928 a series of articles on Cancer and Cancer Research appeared in the *Liverpool Daily Post and Mercury*. These have been reprinted in a handy form and are well worth perusal. In simple language the layman is told of the requirements and methods of research in cancer problems, of its chemical aspects, and of its microscopy and statistics. There is a short account of the prevention of cancer and the available methods of treatment are summarized. We have nothing but praise for the manner in which the information is presented, and for the editorial summary which reviews all the articles. There are many who will welcome its publication; there are some who will question whether any real benefit follows such an issue. However, human mentality demands that the pendulum swing backwards and forwards, and at the present moment the clamour is for diffuseness rather than concentration. After all, it is only the few who realize that the key to pathological growth lies hidden in the genesis of physiological growth.

**Incompatibility in Prescriptions.** By THOMAS STEPHENSON, D.Sc. Ph.C. F.R.S. (Edin.), F.C.S. Pp. 61. Edinburgh: *The Prescriber* Offices. 1929. Price 4s. 6d.—It would be all to the good if a copy of this book were in the hands of every newly-qualified medical man, to whom the writing of a compatible prescription is one of the difficulties least anticipated and most frequently encountered when he leaves the hospital. The book deals effectively with the three main divisions of its subject, viz. Chemical Incompatibility, Physical Incompatibility, Therapeutic Incompatibility. A résumé of each group is first given followed by a survey of the leading examples of each. Particular attention is paid in the first group to cases of incompatibility involving explosive reactions. In addition to the more commonly occurring drugs of the B.P. and B.P.C., such modern preparations as Chloramine T. and Mercurochrome are included. The relative functions of prescriber and pharmacist are indicated by the advice to leave the choice of pill excipients, suspending agents, etc., to the pharmacist. The second half of the book consists of a dictionary of incompatibles, the alphabetical arrangement of the drugs described making it easy for the reader rapidly to turn up any particular drug in question. Under each heading are given: Dosage; Solubilities; Incompatibles. The dictionary includes all types of drugs used, *e.g.* acids, alkaloids, alcohols, ethers, organo-metallic compounds, salts, etc.

**Chronic Otorrhœa.** By A. R. FRIEL, M.D., F.R.C.S.I. Pp. viii., 88. Illustrated. Bristol: John Wright & Sons, Ltd. 1929. Price 6s.—In this small volume the author confines himself to a description of the method of treatment of chronic otorrhœa by zinc ionization. In the first part of the book is given a simplified description of the principles of electrolysis and their method of application in practice. The second part deals with the method of examination and diagnosis, with a special reference to establishing the cause of the chronicity of the aural discharge in individual cases, together with a description of the author's lines of treatment. The remainder outlines the author's scheme of organization of a treatment clinic. In the hands of the author and some of his colleagues the methods laid down appear to have given very excellent results. While the method has its limitations and the results are not markedly superior to older methods of treatment where these can be efficiently carried out, yet in the class of patient dealt with the saving of time effected by the more rapid response to treatment and the less frequent attendances necessary for treatment is an economic factor which is well worthy of wider consideration.

**The Medical Annual, 1929.** Edited by CAREY F. COOMBS, M.D., F.R.C.P., and A. R. SHORT, M.D., B.S., F.R.C.S. Pp. c., 680. Illustrated. Bristol: John Wright & Sons Ltd. 1929. Price 20s.—The present issue well maintains the reputation of this busy man's encyclopædia. It chronicles the chief scientific advances in surgery, radiology, phototherapy and other specialities as recorded in endless scattered publications. It gives long lists of new drugs, books and instruments and directories of societies and institutions for daily reference, and finally, this issue is remarkable for the many hints on difficult points in the treatment of everyday diseases which its general articles provide. Thus it is pointed out that in obstinate hiccough inhalations of $CO_2$ will often give relief, that acne rosacea will often yield to HCl given after meals, that the bleeding in hæmophiliac patients may be arrested by a pad soaked in normal blood after washing away the clots, while bleeding in jaundiced patients can be controlled by calcium if parathyroid is given at the same time. A treatment of hæmatemesis by gelatine given *per os* is also detailed. This last, indeed, is not a new remedy, but excellent as it is, it fell out of use from dread of tetanus after it was shown that hypodermic injections of gelatine could convey that disease. Of course, the tetanus poison is inert when given by the mouth, and it is known that many harmless table jellies contain it, but whether the raw surface of a bleeding ulcer could absorb it is doubtful. By the way, A. R. Short for the same condition recommends an injection of sodium citrate (see p. 199). An article of vital importance to any man who may be summoned before a court of law is that on the care to be taken in making an autopsy, and the omissions and mistakes to be avoided. There have been of late so many unsatisfactory post-mortems recorded in the criminal courts that the warnings in this article are specially opportune.

**Infant Feeding and Nutrition.** By H. B. GLADSTONE, M.D. Pp. xii., 118. London: William Heinemann Ltd. Price 7s. 6d.—This little book is the result of years of experience in infant welfare clinics. It contains a mass of detail and advice in the various methods of feeding babies; indeed, it seems to have this one fault, that, whereas so many alternative plans are submitted, bewildering the reader in their dietetic diversity, no one choice is primarily advocated for a particular type of case; without doubt a vast ingenuity has been displayed in the treatment of the cases quoted, but no precise guide is provided to enable one to determine which course to pursue. The various proprietary foods are critically examined, and much helpful advice is tendered as to their relative merits. Not only is the work intended for the medical profession, but also for those members of the laity engaged in infantile care: hence the various digressions from scientific phraseology to a style assimilable by nurses and others exercising this difficult branch of pediatrics.

**Biological Reversion and Hippocratic Anatomy.** By HUBERT HIGGINS, M.R.C.S. Pp. 149. London: H. K. Lewis & Co. Ltd. 1929. Price 7s. 6d.—"When I use a word," Humpty Dumpty said in rather a scornful tone, "it means just what I choose it to mean—neither more nor less." But he at least explained the meanings he compelled his words to bear, and very properly paid them extra for it. The omission of a like precaution, and the apparent total lack of connection between many paragraphs, make this book very difficult reading, and perhaps explain the author's frequent complaints that his articles languish unpublished in the offices of medical journals. As far as can be ascertained—and there is no index—"biological reversion" means massage, an art, we are led to suppose, entirely unknown to "incurious, indifferent colleagues," and the sole way of salvation. A book that must be seen to be believed.

